# Red and White Meat Intake in Relation to Gut Flora in Obese and Non-Obese Arab Females

**DOI:** 10.3390/foods12020245

**Published:** 2023-01-05

**Authors:** Jinan Almajed, Sara Al-Musharaf, Manal Abudawood, Shaun Sabico, Esra’a A. Aljazairy, Ghadeer S. Aljuraiban

**Affiliations:** 1 Department of Community Health Sciences, College of Applied Medical Sciences, King Saud University, Turki Alawwal street, Riyadh 11451, Saudi Arabia; 2 Department of Clinical Laboratory Sciences, College of Applied Medical Sciences, King Saud University, Riyadh 11433, Saudi Arabia; 3 Chair for Biomarkers of Chronic Diseases, Biochemistry Department, College of Science, King Saud University, Riyadh 11451, Saudi Arabia

**Keywords:** gut flora, red meat, white meat, obesity, inflammatory markers, lipid profile

## Abstract

Background: high meat intake may contribute to several chronic diseases including obesity. However, evidence is insufficient on the relation between red/white meat intake and gut flora among individuals with varying degrees of adiposity. Objective: investigate the association of red/white meat intake with gut flora in Saudi Arabian females with/without obesity. Methods: this observational study involved 92 females with and without obesity (n = 44, 48, respectively) aged 19–25 years. The whole-genome shotgun technique was used to analyze the gut flora. Shannon alpha and Bray–Curtis beta diversity as well as correlation coefficients were used. Results: in the total sample, there were positive correlations between *Actinobacteria, Bacteroides* (*p* ≤ 0.05), *Flavonifractor plautii* (*p* ≤ 0.0001), and total red meat intake. There were also positive correlations between total white meat intake, *Bacteroides*, and *Faecalibacterium prausnitzii* (*p* ≤ 0.05) in the total sample. In the group without obesity, there was a positive correlation between low white meat intake and *Actinobacteria* (*p* = 0.05). In the group with obesity, there was a positive correlation between high white meat intake and *Bacteroides* (*p* ≤ 0.001). Conclusion: our findings suggest that meat intake had an impact on the gut flora of Arab adult females, independent of adiposity. Specific strains identified in this study need further investigation to determine their relation to meat intake and obesity.

## 1. Introduction

In the past decade, global dietary patterns have shifted toward Western-style diets, characterized by higher consumption of refined carbohydrates, added sugars, and animal food sources, including eggs, red meat, processed meat, and white meat [1,2,3]. In 2018, the mean global intake of red meat was ≥50 g/day, having increased by about 88% since 1990 [4]. In Saudi Arabia, the average red meat consumption was high, amounting to ≈50 kg per capita of meat per year in 2017 [5], with an annual growth rate of about 0.24% since 1999 [6]. This increase in meat intake is concurrent with the progressive increase in obesity from 22% in 1990–1993 [7] to 40% in 2017 [8]. High intake of meat has been shown to increase weight gain due to its high energy density and fat content [9]. Compared with other food groups, meat intake was most highly correlated with the prevalence of obesity [10] and other adverse health conditions, such as diabetes [11], cardiovascular disease (CVD) [12], and increased proinflammatory blood markers such as C-reactive protein (CRP), a predictor for CVD risk [13], especially in women [13,14].

Recent studies suggest that diet is one of the most important factors that influence the composition and diversity of the gut flora, eventually modulating the risk of several chronic diseases [15]. The composition of gut bacteria is affected partly by the microorganisms in the ingested food, and partly by the host’s dietary behavior and lifestyle. For example, rats consuming a high-fat diet have a decreased relative abundance of Bacteroidetes and Bifidobacteria and an increased relative abundance of Firmicutes and Proteobacteria [16]. Randomized control trials (RCTs) have reported that meat protein intake correlates with overall microbial diversity [17,18]. However, very few RCTs have investigated the impact of different types of meat on gut flora [17,18,19,20,21], with some revealing an increase in Bacteroides enterotype and a decrease in Firmicutes [17,20] and Clostridium [18] after a high red meat meal. Other RCTs showed that the effect of meat on gut flora may be dependent on the type of meat, i.e., red meat or processed meat [19,21]. It has been suggested that microbial metabolites originating from meat protein, such as short-chain fatty acids, could affect gut flora indirectly by promoting the release of 5-serotonin, leading to raised intestinal motility and ion transport as well as alterations in the gut microbiota composition [22].

Nonetheless, the human gut flora has been observed to differ according to race, ethnicity, and gender, in addition to lifestyle and dietary factors [23,24]. Given the rapid shift in dietary patterns in Saudi Arabia, from a nutrient-rich traditional diet to an energy-dense Western diet [3], and the rising obesity rates, especially in females, it is important to identify the impact of meat intake on gut microbiota composition. To our knowledge, limited evidence is available on the association between the intake of different types of meat and gut flora in the Middle Eastern region. We aim to identify the impact of red/white meat intake on gut flora and the inflammatory marker hs-CRP in Saudi Arabian females with and without obesity. The current study is part of a previous study that aimed to identify the relation between gut microbiota and adiposity. In this study, we focus mainly on dietary factors related to gut flora, specifically meat intake, using the unique whole-genome shotgun (WGS) sequencing technique.

## 2. Materials and Methods

### 2.1. Study Design

The current study is part of a case-control study conducted between January 2019 and March 2020 at the clinic of the College of Applied Medical Sciences in King Saud University, Riyadh, Saudi Arabia, and aiming to identify the relation between gut microbiota and obesity markers. Details on the study design were published previously [25]. Briefly, the sample size was 92, based on the gut microbiota composition and its differences among Saudi females attending university at a 5% significance level and 80% power. The sample was calculated in a similar manner to a previous study [26]. We observed a Firmicutes: Bacteroidetes of 0.9 ± 0.4 in women with normal weight and 1.7 ± 1.7 in women with obesity, with a 95% confidence interval (CI) and 80% power. The participants were randomly recruited via flyers, faculty member assistance, oral presentations, and social media networks. We excluded those who were ≤18 years old, overweight (body mass index (BMI) 25.0–29.9 kg/m^2^), pregnant, or following specific diets (e.g., calorie-restricted diets), as well as those who reported the presence of gastrointestinal diseases in the past eight weeks, endocrine or oncological disease history, psychiatric disorders, anorexia, other medical conditions, and usage of multi-vitamins, vitamin B12, or antibiotics in the past 6 months (n = 193).

After submitting a signed consent form, each participant was given an appointment at the nutrition clinic in the same college and supplied with containers for collecting stool samples that were to be returned on a scheduled visit day. During the visit, demographic, dietary, and anthropometric data were collected, as well as fasting blood samples. Figure S1 represents the number of individuals assessed for participation in the study. The final sample included 92 Saudi female students aged 18 to 25 and classified into obese (BMI ≥ 30 kg/m^2^, n = 44) and non-obese (BMI = 18.50–24.99 kg/m^2^, n = 48). The study protocol was approved by the Institutional Review Board Committee of King Khalid University Hospital at King Saud University (IRB #E-19-3625). Clinical trial registration: URL: https://www.clinicaltrials.gov (accessed on 15 October 2022), unique identifier: NCT05664321.

### 2.2. Anthropometric Measurements

Anthropometric measurements were collected by trained staff using standardized methods. Measurements were recorded twice, and the average results were used for the final analysis. Body weight was measured and recorded to the nearest 0.10 kg and height to the nearest 0.50 cm using an international standard scale (Digital Pearson Scale, ADAM Equipment Inc., Oxford, CT, USA). BMI was calculated by dividing the weight in kilograms by the squared height in meters. BMI was classified into two groups: normal weight (18.50–24.99 kg/m^2^) and obese (≥30 kg/m^2^), using the World Health Organization (WHO) criteria [27].

The waist and hip circumferences were measured using a non-stretchable tape. The waist circumference was measured at the narrowest point between the lowest rib and the umbilicus, and hip circumference at the point of the great trochanter, with the two measurements recorded to the nearest 0.50 cm. The waist–hip ratio (WHR) was calculated by dividing the mean waist circumference by the mean hip circumference [28] and categorized as follows: (i) normal WHR (<0.83) and (ii) high WHR (≥0.83) [28]. Body composition, including fat percentage (BF%) and muscle mass, was measured using a bioelectrical impedance analysis method (770 BIA, Inbody, Seoul, South Korea) [29]. The categories are as follows: (i) normal BF% (≤35%) and (ii) high BF% (>35%) [30].

### 2.3. Dietary Data

Dietary data were collected by trained dietitians during a structured interview. The validated Saudi Food and Drug Authority Food Frequency Questionnaire (FFQ) was used to assess the participants’ dietary intake [31]. The participants were asked to report their frequency of intake of each food item during the past year. The questionnaire was provided in the Arabic language and included 133 food items [31]. Food modules were used to help the participants to determine portion sizes of meat and other foods. ‘Red meat’ referred to beef, camel, goat, or lamb, while ‘white meat’ indicated poultry (chicken, duck, and turkey) and fish [32]. ‘Total meat’ was the total of these two categories. Two 24-h recalls on consecutive days were completed by 20% of the participants to assess the accuracy of the FFQ. Nutrient intake was assessed by the FFQ, and the 24-h recalls correlated well, with a correlation coefficient ranging between 0.50–0.60 for macronutrients and 0.40–0.70 for micronutrients. ESHA Food Processor Software version 11.1 (ESHA Research, Salem, OR, USA) was used for nutrition analysis.

### 2.4. Lipid Profile and Hs-CRP Test

Blood samples were collected after ≥10 h of fasting from the cubital vein into two 5-mL tubes, an ethylenediaminetetraacetic acid tube for the whole blood sample and a gel tube for the serum sample. Serum samples were transferred on the day of collection to the study laboratory and stored at −80 °C to facilitate their availability for further analysis. Lipid profiles were measured using a biochemical analyzer (Konelab, Espoo, Finland). Friedewald’s equation was used to calculate low-density lipoprotein cholesterol (LDL-C) [33]. Serum hs-CRP was measured using commercial enzyme-linked immunosorbent assay kits.

### 2.5. Stool Analysis

Each fecal sample was collected in a clean, dry screw-top container and stored directly at −80 °C. All the samples were then transferred to the study laboratory and kept at −80 °C for further analysis. Later, the DNA was extracted from 0.25 g frozen stool aliquots using the QIAGEN PowerFecal DNA Kit (Catalogue: 12830-50). The purity of the isolated DNA (260/280 ratio) and its concentration was measured using a NanoDrop spectrophotometer (Thermo Fisher Scientific, Waltham, MA, USA). The DNA concentration was ≥1.60. DNA libraries were prepared using the Nextera XT DNA Library Preparation Kit (Illumina) and Nextera Index Kit (Illumina), with a total DNA input of 1 ng. Genomic DNA was fragmented using a proportional amount of Illumina Nextera XT fragmentation enzyme. Combinatory dual indices were added to each sample, followed by 12 cycles of PCR to construct the libraries. The DNA libraries were purified using AMpure magnetic beads (Beckman Coulter) and eluted in QIAGEN EB buffer. The samples were sequenced on an Illumina HiSeq 4000, 2 × 150 bp. DNA extractions were sent to CosmosID (Rockville, MD, USA) to identify the gut microbiota composition at the level of the main microbial phyla by identifying the total bacterial DNA and the Firmicutes, Bacteroidetes, Actinobacteria, Proteobacteria, Verrucomicrobia, and Fusobacteria DNA using the WGS metagenomic sequencing technique.

#### 2.5.1. Bioinformatics Analysis Methods

Unassembled sequencing reads were directly analyzed using the CosmosID bioinformatics platform (CosmosID Inc., Rockville, MD, USA), described elsewhere [34,35,36], for multi-kingdom microbiome analysis, profiling of antibiotic resistance and virulence genes, and quantification of the relative abundance of organisms. The system utilizes a high-performance data-mining k-mer algorithm that rapidly disambiguates millions of short-sequence reads into the discrete genomes engendering the particular sequences. The pipeline has two separable comparators; the first consists of a pre-computation phase for reference databases and the second is a per-sample computation. The input for the pre-computation phase consists of databases of reference genomes, virulence markers, and antimicrobial resistance markers that are continuously curated by CosmosID scientists. The output of the pre-computational phase is a phylogeny tree of microbes together with sets of variable length k-mer fingerprints (biomarkers) uniquely associated with distinct branches and leaves of the tree.

The second per-sample computational phase searches the hundreds of millions of short-sequence reads, or alternatively contigs from draft de novo assemblies, against the fingerprint sets. This query enables the sensitive yet highly precise detection and taxonomic classification of microbial NGS reads. The resulting statistics are analyzed to return the fine-grain taxonomic and relative abundance estimates for the microbial NGS datasets. To exclude false positive identifications, the results are filtered using a filtering threshold based on internal statistical scores that are determined by analyzing a large number of diverse metagenomes. The same approach is applied to enable the sensitive and accurate detection of genetic markers for virulence and for resistance to antibiotics.

#### 2.5.2. Alpha Diversity Boxplots (with Wilcoxon Rank-Sum)

Alpha diversity boxplots were calculated from the phylum, genus, species, and strain-level abundance score matrices from the CosmosID-HUB analysis. Chao, Simpson, and Shannon alpha diversity metrics were calculated in R using the R package vegan [37,38]. Wilcoxon Rank-Sum tests were performed between groups using the R package ggsignif [39]. Boxplots with overlaid significance in *p*-value format were generated using the R package ggpubr [40].

#### 2.5.3. Beta Diversity PCoA (with PERMANOVA)

Beta diversity principal coordinate analyses were calculated from phylum, genus, species, and strain-level matrices for bacteria from CosmosID-HUB. Bray–Curtis dissimilarity was calculated in R using the vegan package with the function vegdist, and PCoA tables were generated using ape’s function pcoa [41]. PERMANOVA tests for each distance matrix were generated using vegan’s [38] function adonis2, and beta dispersion was calculated and compared using the anova method for the betadisper function from vegan [38]. Plots were visualized using the R package ggpubr [40].

### 2.6. Statistical Analysis

Quantitative variables were tested for normality prior to the analysis. The normality of the variables was assessed visually using a histogram and Q-Q plot and/or by evaluating skewness and kurtosis. The independent samples *t*-test was used for continuous variables and outcomes. Nonparametric tests were used for variables that were not normally distributed and that skewed toward one side. To identify the impact of meat by type, we divided the participants into distinct categories, i.e., high and low intake, using the median of each type of meat as the cut-off point. For the intake of white meat, the non-obese group was considered to have high white meat (HWM, n = 32) if the intake was >34 g/1000 kcal and low white meat (LWM, n = 16) if the intake was ≤34 g/1000 kcal. The obese group was stratified into high (HWM, n = 28) if the intake was >25 g/1000 kcal and low (LWM, n = 16) if the intake was ≤25 g/1000 kcal.

The non-obese group was also stratified according to red meat intake into high red meat intake (HRM, n = 21) if intake was >12 g /1000 kcal and low red meat intake (LRM, n = 27) if intake was ≤12 g/1000 kcal. The obese group was considered to have high red meat intake (HRM, n = 14) if intake was >16 g/1000 kcal and low red meat intake (LRM, n = 30) if the red meat intake was ≤16 g/1000 kcal.

A Pearson correlation coefficient was used to identify the correlations between meat (total and by type) and gut flora. All non-normal variables were transformed prior to parametric testing. A *p*-value of <0.05 and a 95% CI were used to report estimated statistical significance. Furthermore, a Benjamini-Hochberg critical value for a false discovery rate of 0.25 was computed for all correlations. The analysis was carried out using IBM SPSS Statistics for Windows (version 24, IBM Corp., Armonk, NY, USA).

To identify the distribution of species abundances and similarities between groups, the CosmosID application was used to examine the relationship between gut flora and different levels and types of meat intake in the obese and non-obese groups, and by body fat percentage and waist to hip ratio stratification. The Shannon test was used for alpha diversity, which represents the distribution of species abundances in a given sample as a number that depends on species evenness and richness, concentrating on community variation within a single sample [42]. For beta diversity, Bray–Curtis was used to measure the similarity or dissimilarity between samples [42].

## 3. Results

### 3.1. Characteristics of Participants

Blood biochemical data, including total cholesterol (TC), HDL-C, LDL-C, total cholesterol/HDL ratio, triglyceride (TG), and hs-CRP, were significantly higher in the obese group compared with the non-obese group (*p <* 0.05) (Table 1). Additionally, total red meat intake (g/1000 kcal) was significantly higher in the obese group compared with the non-obese group (*p <* 0.05) (Table 1).

### 3.2. Correlation between Gut Flora and Meat Intake

Among the total participants, there were positive correlations between *Actinobacteria, Bacteroides* (unidentified species) (*p* ≤ 0.05), *Flavonifractor plautii* (*p* ≤ 0.0001), and total red meat intake (Table 2). For total white meat intake, there were positive correlations between *Bacteroides* (unidentified species) and *Faecalibacterium Prausnitzii* (*p* ≤ 0.05).

#### 3.2.1. White Meat Intake and Gut Flora

In the non-obese group, there was a positive correlation between LWM intake, *Actinobacteria* (*p* = 0.05), and *Bifidobacterium longum* (*p* = 0.02) (Table 3). There was also a positive correlation between HWM intake and *Bacteroidetes* (*p* = 0.03), *Flavonifractor plautii* (*p* ≤ 0.001), and *Clostridium Bolteae* (*p* ≤ 0.001), and an inverse correlation with *Firmicutes* (*p* = 0.04).

In the obese group, there was a positive correlation between HWM intake and *Bacteroides (unidentified species)* (*p* ≤ 0.001), *Faecalibacterium Prausnitzii* (*p* = 0.04), and *Clostridium difficile* (*p* = 0.03) (Table 3).

#### 3.2.2. Red Meat Intake and Gut Flora

In the non-obese group, HRM intake correlated positively with *Flavonifractor plautii* (*p* ≤ 0.001) and inversely with *Akkermansia muciniphila* (*p* = 0.05) (Table 4).

In the obese group, there were no significant correlations between HRM intake and gut flora (*p* > 0.05) (Table 4).

#### 3.2.3. Correlation between Gut Flora and Hs-CRP and Lipid Profile

In the total sample, hs-CRP was inversely correlated with *Bifidobacterium adolescentis* (*p* = 0.04) (Table S1)**.** Lipid profile, including TC, HDL-C, LDL-C, TC/HDL ratio, and TG did not show significant correlations with gut bacteria.

#### 3.2.4. Meat Intake and Gut Flora (Alpha and Beta Diversity)

**Figure 1:**
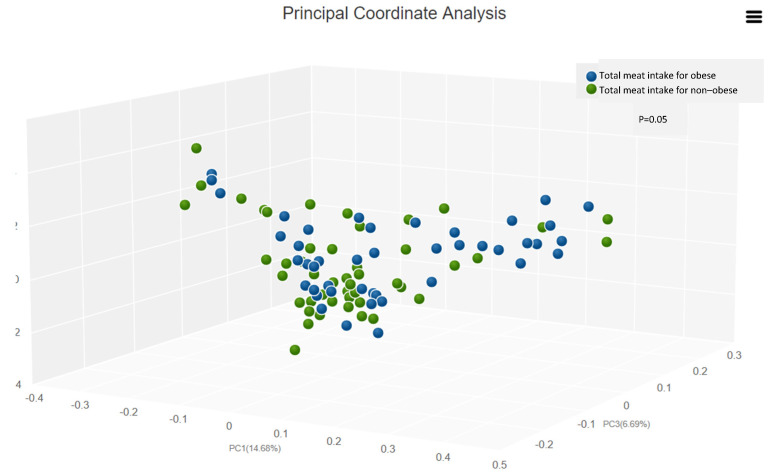
Total meat intake across obese and non-obese groups (beta diversity, Bray–Curtis).

Gut flora diversity results showed no significant differences in alpha diversity (*p* = 0.09) between total meat intake in the obese and non-obese groups (Figure S1). However, there was a significant difference in beta diversity (*p* = 0.05) between total meat intake in the obese and non-obese groups (Figure 1). Moreover, there were no significant differences in Alpha diversity (*p* > 0.05) between high and low white meat intake in the obese and non-obese groups (Figure S2). There were no significant differences in Alpha diversity (*p* > 0.05) between high and low red meat intake in the obese and non-obese groups (Figure S3).

## 4. Discussion

### 4.1. Main Findings

In this study, we found significant correlations between types of meat intake and certain gut bacteria among the total participants, independent of adiposity. We also found significant correlations between types of meat intake and specific gut bacteria which differed among obese and non-obese groups. There was a significant difference in beta diversity between total meat intake in the obese and non-obese groups, but not in alpha diversity. Further, we found that the hs-CRP level correlated negatively with *Bifidobacterium adolescentis*. No significant relationship was observed between lipid profile and gut flora.

### 4.2. Comparison with Previous Studies

Our findings show that Actinobacteria, Bacteroides (unidentified species), and Flavonifractor plautii correlated positively with total red meat intake, while both Bacteroides (unidentified species) and Faecalibacterium Prausnitzii correlated positively with total white meat intake among the total participants, indicating that these associations are independent of adiposity.

A previous cross-sectional study that included 98 healthy volunteers aged between 2 and 50 years showed that *Bacteroides* correlated directly with animal protein, implying that meat consumption is characterized by this enterotype. Furthermore, *Bacteroidetes* and *Actinobacteria* phyla were positively associated with fat intake, whereas *Firmicutes* and *Proteobacteria* showed inverse associations [20].

To get a clearer understanding of the relation between gut flora and different types of meat relative to obesity, we stratified the participants by both adiposity level and type of meat. In the non-obese group, *Bifidobacterium longum* was positively correlated with LWM. These findings are in agreement with a study by Reyeset et al., which reported that an abundance of *Bifidobacterium longum* was positively correlated with unsaturated fat intake, and that it was more abundant in the lean group than in the overweight and obese groups [43]. We also found that in the non-obese group, *Flavonifractor plautii* was positively correlated with both HWM and HRM intakes. In contrast, a previous RCT that determined the influence of fried meat intake on gut microbiota showed that fried meat intake reduced microbial richness and Flavonifractor abundance [44]. However, the cooking method, such as frying or grilling, may itself have influenced the level of this bacteria [45,46]. We also observed that for the non-obese group there was an inverse correlation between HRM intake and *Akkermansia muciniphila*. A previous animal study reported differences in the responses of *Akkermansia muciniphila* to the intake of two different types of protein (chicken protein and soy protein) [47], with the chicken protein-based diet maintaining the *Akkermansia muciniphila* level in the gut and the soy protein-based diet decreasing the abundance of this bacteria [47]. Nevertheless, several human and animal studies have reported that many types of food and medications may affect *Akkermansia muciniphila* [48]. Thus, more studies are needed to identify the mechanism behind the effect of red meat on *Akkermansia muciniphila* while considering other factors that might influence this bacterium. Furthermore, in the non-obese group, there was a positive correlation between LWM intake and *Actinobacteria*, in contrast to an animal study in which rats fed a high amount of chicken protein had a greater abundance of *Actinobacteria* than other protein groups [49].

In the obese group, we found that HWM intake was positively correlated with *Clostridioides difficile*, a finding consistent with a study by Heise et al. (2021) in which 364 different fresh poultry products were screened and 15.80% of samples were shown to have positive levels of *Clostridioides difficile* [50]. Further, we observed that in the obese group HWM intake was directly correlated with *Faecalibacterium prausnitzii*. However, a previous prospective observational study showed that following a ketogenic diet that included protein from animal sources such as meat and poultry for three months did not alter *Faecalibacterium prausnitzii* levels [51]. *Faecalibacterium prausnitzii* is one of the main butyrate producers in the intestine and it plays a crucial role in gut physiology and host wellbeing [52]. It is the main energy source for colonocytes and confers protective properties against colorectal cancer and inflammatory bowel diseases. However, *Faecalibacterium prausnitzii* levels have depleted considerably over the past few years [52]. Further investigations into which gut factors modulate its presence are warranted.

From a gut diversity perspective, our study showed that significant differences in gut flora diversity were found only in beta diversity between varying amounts and types of meats. Alpha and beta diversity were measured to observe the richness and variability of gut microbiota composition, respectively [42]. The results of the current study are consistent with a previous study by David et al. (2014), which revealed that no significant differences in alpha diversity were detected between plant- or animal-based diets. However, there was a significant difference in beta diversity that was specific to animal-based diets [17]. Nevertheless, several other factors may influence gut microbes more than diet effects [53]. For example, two large-scale observational population studies recognized 69 and 126 factors related to inter-individual and health traits associated with the gut microbiota [53,54]. Obesity was determined to be one of the important influential factors that affect gut microbiota [55], and low gut microbiota diversity and richness were correlated with an increased risk of obesity [56].

Regarding hs-CRP and gut flora, our results showed a significant inverse correlation between the hs-CRP level and *Bifidobacterium adolescentis*. A study by Rajkumar et al. (2014) showed that participants with a high hs-CPR level (>3 mg/L) had significantly lower *bifidobacteria* and *lactobacilli* compared with those who had a low hs-CRP level (<3 mg/L) [57]. Moreover, a systematic review of 14 observational studies that investigated the association between gut microbiota and hs-CPR level showed that inflammatory markers such as hs-CRP and IL-6 were inversely correlated with *Bifidobacterium, Faecalibacterium, Ruminococcus,* and *Prevotella* [58]. The inverse correlation between the hs-CRP level and *Bifidobacterium adolescentis* may be explained by the association of some *Bifidobacterium* taxa with reduced systemic inflammation [59,60]. Further, our study showed no significant correlation between lipid profile and gut flora in contrast with other studies that showed significant correlations between different gut flora and lipid profiles [61,62], which may be because specific operational taxonomic units reported to be associated with lipid profiles did not exist in the participants in our study. Moreover, other factors may influence the lipid profile besides the gut flora, such as age, sex, and genetics [61].

The main limitation of this study is the generalizability of these results, as the study population included only females with a specific age range. Moreover, this study is based on observations, and therefore causality is not possible. Furthermore, even with the extensive measures used in the data collection process, there was still a possibility of recall bias, instrument error, and confirmation bias. One of the study’s strengths is its use of the WGS technique, the gold standard method for identifying the gut flora composition, showing the highest sensitivity compared with other techniques [63]. Moreover, the design of the study allowed us to identify the differences in the intake of various amounts and types of meat in relation to gut flora. Additionally, to our knowledge, our work is the first to examine associations between types of meat intake, gut flora, lipid profile, and hs-CRP in females in the Middle Eastern region. Furthermore, precautions such as repeated measurements, trained staff, and interview-based multi-pass dietary assessment methods were used to increase the accuracy and precision of the data collection process.

## 5. Conclusions

In conclusion, our findings suggest that certain bacteria are correlated with meat intake (by type of meat), independent of adiposity. We also found significant correlations between types of meat intake and specific gut bacteria which differed among obese and non-obese groups. Further investigations are needed to explore the correlations we reported, which are not explained in the literature, between meat intake and different gut bacteria, including *Flavonifractorplautii, Actinobacteria, Bifidobacteriumlongum, Clostridiumbolteae, Firmicutes, Faecalibacterium prausnitzii, Clostridioides difficile, Akkermansia muciniphila*, and *Bacteroides* with unidentified species. Moreover, the relationship between gut flora and meat intake can differ in beta diversity between obese and non-obese groups, but not in alpha diversity. Future studies with a larger number of participants of different ages, from both genders, and from various regions are recommended.

## Figures and Tables

**Table 1:** Characteristics of obese and non-obese groups, n = 92 ^1^.

Characteristics	Total (*n* = 92)	Non-Obese (BMI 18.50–24.99) (*n* = 48)	Obese (BMI ≥ 30) (*n* = 44)	*p*-Value
Age (years) (mean ± SD)	21.10 ± 1.50	20.60 ± 1.10	21.60 ± 1.70	<0.001
BMI (kg/m^2^)	28.50 ± 8.00	21.70 ± 1.90	36.00 ± 4.70	<0.001
Waist to hip ratio (cm)	0.70 ± 0.10	0.70 ± 0.10	0.80 ± 0.10	0.01
Fat (kg)	42.50 ± 9.40	34.80 ± 5.50	51.10 ± 3.30	<0.001
Skeletal muscle mass (kg)	21.00 ± 3.50	18.60 ± 2.20	23.60 ± 2.60	<0.001
Total white meat (g/1000 kcal)	32 (19–41)	34 (26–41)	25 (16–40)	0.11
Total red meat (g/1000 kcal)	15 (9–19)	12 (4–16)	16 (13–21)	<0.001
LWM (%)	35	33	36	0.01
HWM (%)	65	67	64	
LRM (%)	62	56	68	
HRM (%)	38	44	32	
Biochemical Measurements				
Total Cholesterol (mmol/L)	4.10 ± 1.50	3.60 ± 1.70	4.50 ± 1.00	0.01
HDL-Cholesterol (mmol/L)	1.00 ± 0.30	0.90 ± 0.40	1.00 ± 0.30	0.24
LDL-Cholesterol (mmol/L)	2.90 ± 1.30	2.60 ± 1.50	3.30 ± 1.00	0.01
Total cholesterol/HDL ratio	4.30 ± 1.70	3.90 ± 1.70	4.70 ± 1.70	0.05
Triglyceride (mmol/L)	0.70 (0.50–1.00)	0.50 (0.40–0.70)	1.00 (0.80–1.10)	<0.001
High-sensitivity C-reactive Protein (ng/mL)	1473.90 (787.10–7635.60)	1031.20 (553–1514)	6385.40 (1235–12,988)	<0.001

^1^ Data are presented as mean ± SD for normal variables, median (1st quartile–3rd quartile) for non-normal variables, and N (%) for categorical variables. BMI: body mass index; HDL: high-density lipoprotein; HRM: high red meat intake; HWM: high white meat intake; LDL: low-density lipoprotein; LRM: low red meat intake; LWM: low white meat intake.

**Table 2:** Correlations between gut flora by type of meat intake among total participants ^1^.

Gut Flora	Total White Meat (g/1000 kcal)			Total Red Meat (g/1000 kcal)		
	R	*p*	(i/m) Q	R	*p*	(i/m) Q
*Flavonifractor plautii*	−0.04	0.70	0.18	0.31	<0.0001	0.01
*Bacteroides (unidentified species)*	0.21	0.05	0.01	0.23	0.04	0.03
*Actinobacteria*	−0.11	0.31	0.06	0.22	0.05	0.04
*Faecalibacterium Prausnitzii*	0.22	0.05	0.03	0.18	0.07	0.06
*Bifidobacterium adolescentis*	−0.09	0.42	0.12	0.15	0.15	0.07
*Fusobacteria*	0.06	0.61	0.16	−0.11	0.32	0.09
*Bifidobacterium longum*	−0.11	0.31	0.07	0.09	0.41	0.10
*Proteobacteria*	−0.02	0.87	0.21	0.08	0.46	0.12
*Clostridium Bolteae*	0.08	0.45	0.15	0.08	0.47	0.13
*Firmicutes*	−0.08	0.45	0.13	−0.07	0.49	0.15
*Verrucomicrobia*	0.01	0.90	0.24	−0.04	0.74	0.16
*Akkermansia muciniphila*	0.01	0.89	0.22	−0.03	0.75	0.18
*F:B ratio*	−0.01	0.96	0.25	−0.02	0.83	0.19
*Bacteroides uniformis*	−0.15	0.15	0.04	−0.02	0.85	0.21
*Clostridium difficile*	0.04	0.71	0.19	0.02	0.86	0.22
*Bacteria (unidentified phylum)*	−0.1	0.36	0.09	0.01	0.94	0.24
*Bacteroidetes*	0.11	0.37	0.10	0.01	0.95	0.25

^1^ Data are presented as Pearson correlation coefficient adjusted for age. F:B: Firmicutes to Bacteroidetes; (i/m) Q: Benjamini-Hochberg critical value for a false discovery rate of 0.25.

**Table 3:** Correlations between gut flora and white meat intake in obese and non-obese groups ^1^.

Gut Flora	Non-Obese						Obese					
	LWM			HWM			LWM			HWM		
	R	*p*	(i/m) Q	R	*p*	(i/m) Q	R	*p*	(i/m) Q	R	*p*	(i/m) Q
*Actinobacteria*	0.42	0.05	0.03	−0.21	0.34	0.13	0.06	0.76	0.18	−0.22	0.45	0.12
*Bifidobacterium longum*	0.48	0.02	0.02	0.01	0.96	0.25	−0.04	0.84	0.22	−0.28	0.33	0.09
*Bacteroidetes*	−0.23	0.29	0.11	0.45	0.03	0.05	−0.13	0.53	0.10	0.03	0.91	0.25
*Bacteria (unidentified phylum)*	−0.09	0.69	0.23	−0.20	0.37	0.14	0.17	0.40	0.03	0.43	0.12	0.06
*F:B ratio*	0.16	0.46	0.20	−0.25	0.26	0.09	0.12	0.56	0.12	0.06	0.84	0.19
*Clostridium Bolteae*	0.25	0.26	0.09	0.60	<0.001	0.03	0.04	0.86	0.24	−0.06	0.85	0.21
*Firmicutes*	0.09	0.67	0.22	−0.43	0.04	0.06	0.12	0.57	0.15	0.05	0.87	0.24
*Flavonifractor plautii*	0.27	0.21	0.08	0.60	<0.001	0.02	0.02	0.90	0.25	−0.27	0.35	0.10
*Clostridium difficile*	0.23	0.29	0.13	0.03	0.90	0.23	−0.05	0.80	0.21	0.67	0.03	0.03
*Bacteroides uniformis*	0.18	0.41	0.19	0.15	0.49	0.20	−0.05	0.79	0.19	−0.43	0.12	0.07
*Bacteroides (unidentified species)*	−0.37	0.09	0.05	0.12	0.58	0.22	0.21	0.29	0.01	0.85	<0.001	0.01
*Faecalibacterium Prausnitzii*	0.19	0.38	0.14	−0.15	0.49	0.17	−0.10	0.63	0.16	0.55	0.04	0.04
*Bifidobacterium adolescentis*	0.29	0.17	0.06	−0.23	0.28	0.11	0.12	0.56	0.13	−0.09	0.77	0.16
*Proteobacteria*	0.07	0.76	0.25	−0.33	0.13	0.08	0.16	0.43	0.07	−0.15	0.61	0.13
*Fusobacteria*	−	−		−	−		0.13	0.51	0.09	−0.05	0.86	0.22
*Verrucomicrobia*	0.18	0.41	0.16	−0.15	0.49	0.19	−0.17	0.40	0.06	0.08	0.78	0.18
*Akkermansia muciniphila*	0.18	0.41	0.17	−0.18	0.41	0.16	−0.17	0.40	0.04	0.09	0.77	0.15

^1^ Data are presented as Pearson correlation coefficients adjusted for age. F:B: Firmicutes to Bacteroidetes; HWM: high white meat intake; LWM: low white meat intake; (i/m) Q: Benjamini-Hochberg critical value for a false discovery rate of 0.25. High white meat intake is >25 g/1000 kcal for the obese group, low white meat intake is ≤25 g/1000 kcal for the obese group, high white meat intake is >34 g/1000 kcal for the non-obese group, and low white meat intake is ≤34 g/1000 kcal for the non-obese group. The hyphens in cells indicate that gut flora is not present in these categories or that the number of observations for this group is not high enough to report a correlation coefficient.

**Table 4:** Correlations between gut flora and red meat intake in obese and non-obese groups ^1^.

Gut Flora	Non-Obese						Obese					
	LRM			HRM			LRM			HRM		
	R	*p*	(i/m) Q	R	*p*	(i/m) Q	R	*p*	(i/m) Q	R	*p*	(i/m) Q
*Actinobacteria*	−0.16	0.48	0.05	0.01	0.95	0.23	0.27	0.14	0.04	0.55	0.10	0.02
*Bifidobacterium longum*	−0.13	0.57	0.06	−0.16	0.44	0.13	0.30	0.10	0.01	0.46	0.18	0.04
*Bacteroidetes*	0.04	0.85	0.19	0.05	0.83	0.19	0.06	0.74	0.19	−0.35	0.32	0.05
*Bacteria (unidentified phylum)*	0.00	0.99	0.23	0.21	0.32	0.08	0.10	0.59	0.18	0.34	0.33	0.07
*F:B ratio*	−0.02	0.94	0.22	−0.01	0.98	0.25	−0.15	0.41	0.15	0.31	0.38	0.09
*Clostridium Bolteae*	−0.04	0.87	0.20	0.21	0.32	0.06	−0.14	0.45	0.16	−0.29	0.41	0.11
*Firmicutes*	−0.07	0.77	0.17	−0.05	0.82	0.17	−0.16	0.38	0.12	0.20	0.59	0.13
*Flavonifractor plautii*	−0.08	0.73	0.16	0.74	0.00	0.02	−0.06	0.75	0.21	−0.17	0.64	0.14
*Clostridium difficile*	−0.08	0.72	0.14	−0.19	0.37	0.09	0.01	0.95	0.25	−0.17	0.64	0.16
*Bacteroides uniformis*	0.01	0.99	0.25	−0.19	0.38	0.11	−0.04	0.82	0.22	0.14	0.69	0.18
*Bacteroides (unidentified species)*	−0.20	0.38	0.03	0.07	0.74	0.16	−0.04	0.84	0.24	−0.13	0.72	0.20
*Faecalibacterium Prausnitzii*	−0.09	0.69	0.13	−0.15	0.49	0.14	0.23	0.20	0.07	0.12	0.75	0.21
*Bifidobacterium adolescentis*	−0.13	0.57	0.08	0.03	0.90	0.22	0.26	0.16	0.06	0.07	0.85	0.23
*Proteobacteria*	0.28	0.20	0.02	−0.04	0.84	0.20	0.16	0.40	0.13	0.02	0.96	0.25
*Fusobacteria*	-	-		-	-		−0.30	0.10	0.03	-	-	-
*Verrucomicrobia*	0.09	0.68	0.11	−0.37	0.08	0.05	−0.18	0.34	0.09	-	-	-
*Akkermansia muciniphila*	0.09	0.68	0.09	−0.40	0.05	0.03	−0.17	0.37	0.10	-	-	-

^1^ Data are presented as Pearson correlation coefficients adjusted for age. F:B: Firmicutes to Bacteroidetes; HRM: high red meat intake; (i/m) Q: Benjamini-Hochberg critical value for a false discovery rate of 0.25; LRM: low red meat intake. High red meat intake is > 11 g/1000 kcal for the obese group, low red meat intake is ≤ 11 g/1000 kcal for the obese group, high red meat intake is > 16 g/1000 kcal for the non-obese group, and low red meat intake is ≤ 16 g/1000 kcal for the non-obese group. The hyphens in cells indicate that gut flora is not present in these categories or that the number of observations for this group is not high enough to report a correlation coefficient.

## Data Availability

The data presented in this study are available on request from the corresponding author.

## Supplementary Materials

The following supporting information can be downloaded at: https://www.mdpi.com/article/10.3390/foods12020245/s1, Figure S1: Total meat intake across obese and non-obese groups (alpha diversity-Shanon); Figure S2: High and low white meat intake across cases and controls (Alpha diversity- Shannon); Figure S3: High and low red meat intake across cases and controls (Alpha diversity- Shannon). Table S1: Correlation between gut flora and lipid profile.
