# Supplementary material for: Red and White Meat Intake in Relation to Gut Flora in Obese and Non-Obese Arab Females
Source: Foods. 2023 Jan 5;12(2):245. doi: 10.3390/foods12020245 (PMC9857880; doi:10.3390/foods12020245)
Supplement: Supplementary file 1 [file foods-12-00245-s001.zip › foods-2032815-supplementary.pdf]

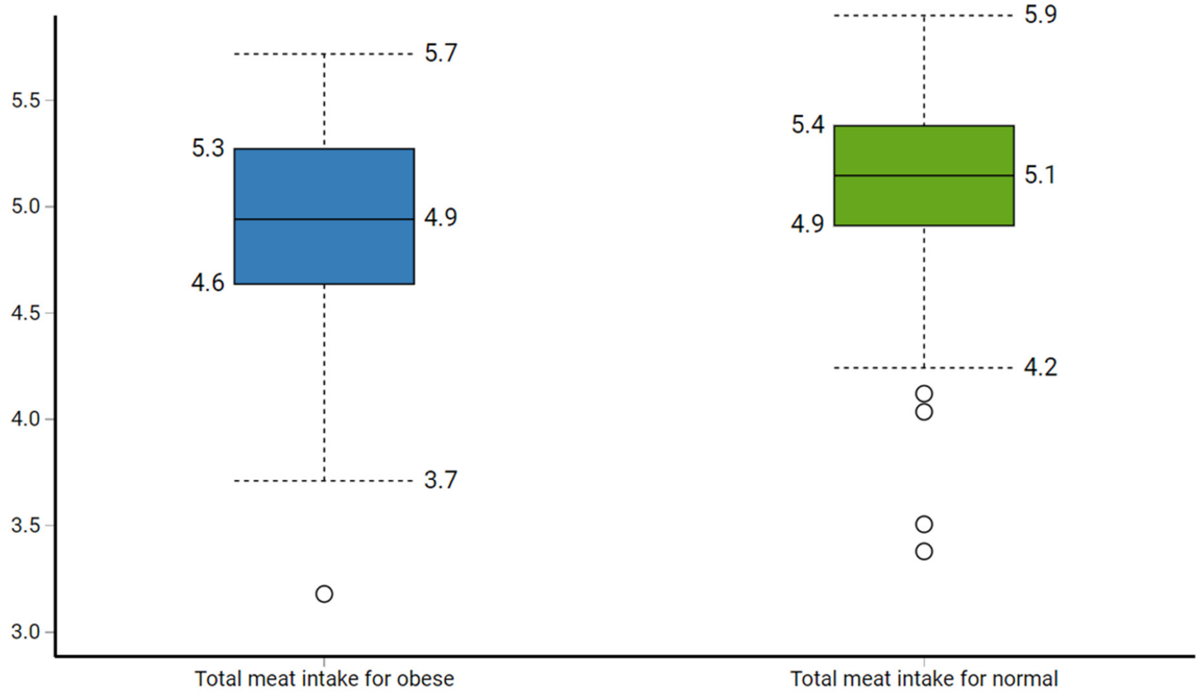

Figure S1. Total meat intake across obese and non-obese groups (alpha diversity-Shanon)

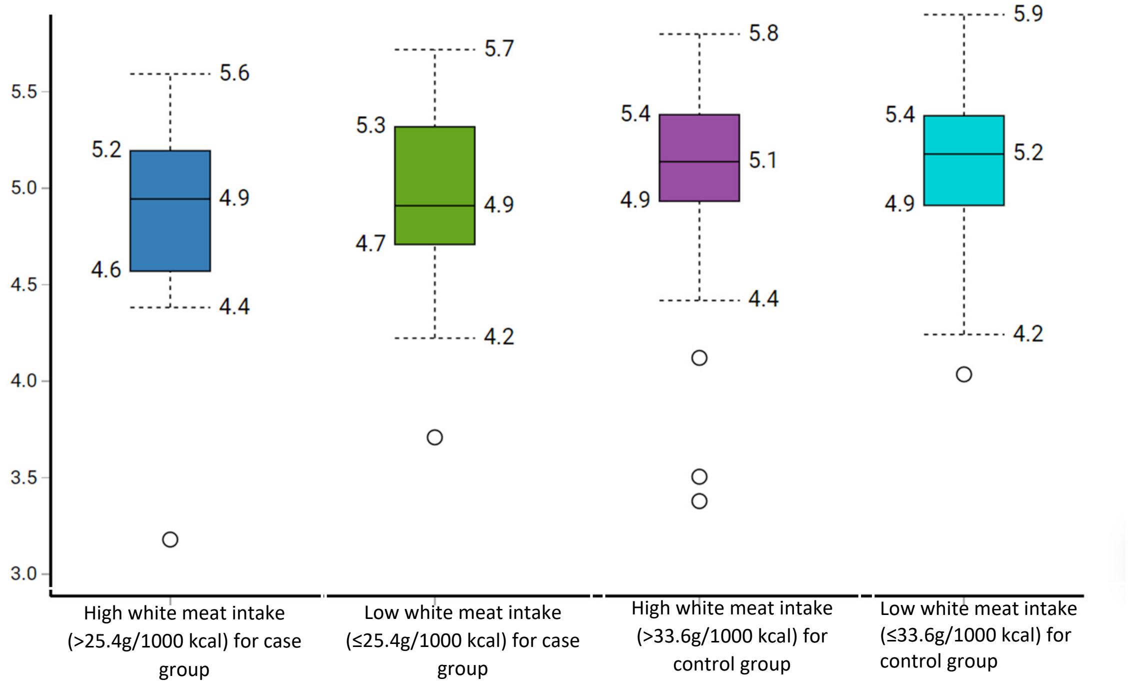

Cohorts	P-value
High white meat intake for case group ↔ Low white meat intake for case group	0.925
High white meat intake for case group ↔ High white meat intake for control group	0.253
High white meat intake for case group ↔ Low white meat intake for control group	0.129
Low white meat intake for case group ↔ High white meat intake for control group	0.455
Low white meat intake for case group ↔ Low white meat intake for control group	0.195
High white meat intake for control group ↔ Low white meat intake for control group	0.726

Figure S2: High and low white meat intake across cases and controls (Alpha diversity-Shannon)

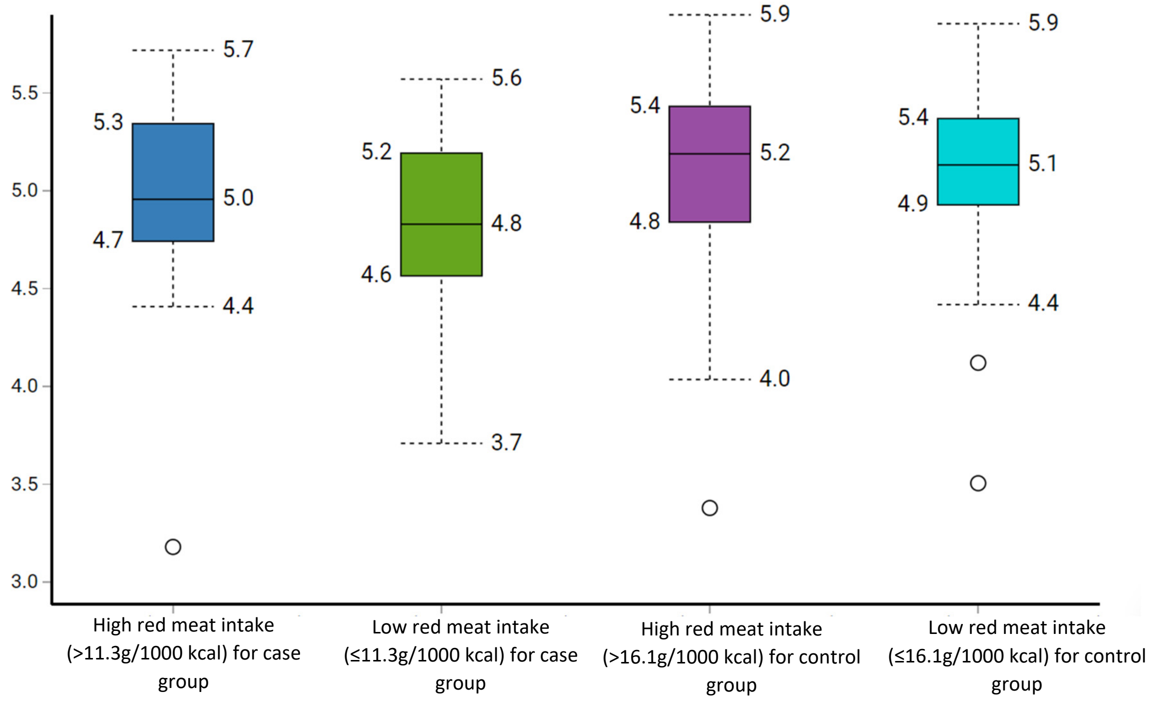

Cohorts	P-value
High red meat intake for case group ↔ Low red meat intake for case group	0.270
High red meat intake for case group ↔ High red meat intake for control group	0.388
High red meat intake for case group ↔ Low red meat intake for control group	0.551
Low red meat intake for case group ↔ High red meat intake for control group	0.097
Low red meat intake for case group ↔ Low red meat intake for control group	0.110
High red meat intake for control group ↔ Low red meat intake for control group	0.828

Figure S3: High and low red meat intake across cases and controls (Alpha diversity-Shannon)
